# Genetic Diversity of Croatian Common Bean Landraces

**DOI:** 10.3389/fpls.2017.00604

**Published:** 2017-04-20

**Authors:** Klaudija Carović-Stanko, Zlatko Liber, Monika Vidak, Ana Barešić, Martina Grdiša, Boris Lazarević, Zlatko Šatović

**Affiliations:** ^1^Department of Seed Science and Technology, Faculty of Agriculture, University of ZagrebZagreb, Croatia; ^2^Centre of Excellence for Biodiversity and Molecular Plant Breeding (CroP-BioDiv)Zagreb, Croatia; ^3^Department of Botany, Faculty of Science, University of ZagrebZagreb, Croatia; ^4^Department of Plant Nutrition, Faculty of Agriculture, University of ZagrebZagreb, Croatia

**Keywords:** common bean, landrace, origin, phaseolin type, microsatellite markers

## Abstract

In Croatia, the majority of the common bean production is based on local landraces, grown by small-scale farmers in low input production systems. Landraces are adapted to the specific growing conditions and agro-environments and show a great morphological diversity. These local landraces are in danger of genetic erosion caused by complex socio-economic changes in rural communities. The low profitability of farms and their small size, the advanced age of farmers and the replacement of traditional landraces with modern bean cultivars and/or other more profitable crops have been identified as the major factors affecting genetic erosion. Three hundred accessions belonging to most widely used landraces were evaluated by phaseolin genotyping and microsatellite marker analysis. A total of 183 different multi-locus genotypes in the panel of 300 accessions were revealed using 26 microsatellite markers. Out of 183 accessions, 27.32% were of Mesoamerican origin, 68.31% of Andean, while 4.37% of accessions represented putative hybrids between gene pools. Accessions of Andean origin were further classified into phaseolin type II (“H” or “C”) and III (“T”), the latter being more frequent. A model-based cluster analysis based on microsatellite markers revealed the presence of three clusters in congruence with the results of phaseolin type analysis.

## Introduction

Common bean (*Phaseolus vulgaris* L.) is a valuable legume for human consumption worldwide, being an important source of high quality proteins, carbohydrates, vitamins, minerals, dietary fiber, phytonutrients (flavonoids, lignins, phytosterols) and antioxidants (Cardador-Martínez et al., 2002; Reynoso-Camacho et al., 2006). Many of these compounds have important beneficial effects on human health, therefore, common bean has considerable potential as a functional food.

Common bean was introduced into Europe from mutually independent domestication centers, Central and South America, where the Mesoamerican and the Andean cultivated gene pools originated (Gepts and Debouck, 1991). Common bean landraces originated from these two gene pools were introduced into Europe at different times. The Mesoamerican common bean landraces probably arrived in Europe through Spain and Portugal in 1,506, and the Andean in the same way in 1,528, after the exploration of Peru by Pizarro (Gioia et al., 2013). Subsequent spread of common bean landraces throughout Europe was very complex with several introductions from various regions of the Americas, combined with frequent exchanges between European and other Mediterranean countries (Papa et al., 2006). The common bean is distributed in Europe, Asia and Africa, where it presents similarities to Andean and Mesoamerican genepools or forms hybrids between both genepools (Chávez-Servia et al., 2016). Gene flow between domesticated and wild beans led to substantial introgression of alleles from the domesticated gene pool into the wild gene pool and vice versa (Pathania et al., 2014). Gene pool diversity has been validated using various marker systems including seed size, plant morphology, phaseolin seed protein patterns, allozymes and molecular markers (Asfaw et al., 2009). Europe, Brazil, central-eastern and southern Africa and China have been suggested as the secondary centers of diversification for common bean (Bellucci et al., 2014).

It has been proven that phaseolin, the major seed storage protein of common bean, is an important molecular marker in studies of genetic diversity and evolution of common bean populations due to its functional and structural properties (De la Fuente et al., 2012). Two gene pools of domestication are distinguished in the species and are characterized by morphological differences as well as by phaseolin types. Predominant phaseolyne types are “S” (Mesoamerican) or “T”/“C”/“H” (Andean), (Raggi et al., 2013). According to studies based on phaseolin analyses the Andean gene poll of common bean is always prevalent in Europe, between 66 and 76% (Lioi, 1989; Logozzo et al., 2007; Angioi et al., 2010).

In Europe in recent decades, in response to market demands, landraces have progressively been replaced by improved cultivars but some studies have shown that many landraces survive on-farm in marginal areas of several European countries (Lioi et al., 2005). In Croatia the production of common bean is based on landraces which are adapted to the specific growing conditions and agro-environments which display high levels of morphological diversity (Čupić et al., 2012). Landraces are traditionally grown in low-input production systems. Preservation of the genetic diversity that is held by small-scale farmers could provide important sources of genetic resistance for plant breeders, as they are likely to contain alleles for local adaptations, disease resistance, and tolerance to the principal climate adversities in the region. However, in recent years, landraces are in danger of genetic erosion caused by complex socio-economic changes in rural communities (the low profitability of farms, their small size, and the advanced age of farmers, the replacement of traditional landraces with modern bean cultivars and/or other more profitable crops; FAO, 2008).

In order to depict the origin and diversity of common bean landraces, it is necessary to conduct analyses at the morphological and genetic level. Therefore, the aim of this research was the assessment of genetic diversity and structure of Croatian common bean landraces using microsatellites and the determination of their origin by phaseolin marker analysis. The results were discussed in a broader, European context.

## Materials and methods

### Plant material

**Figure 1:**
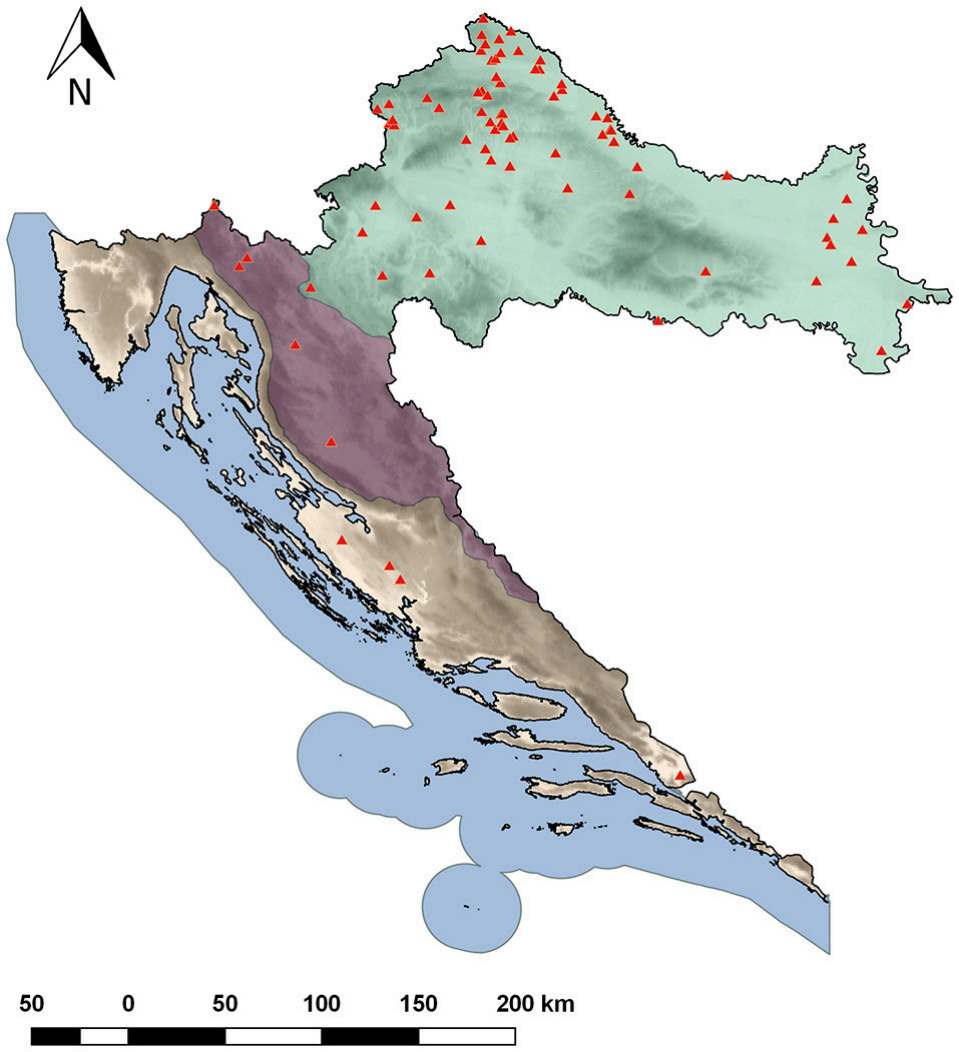
**Common bean sampling locations in Croatia**.

Three-hundred accessions of common bean landraces were collected from diverse geographical regions of Croatia (Figure 1) and grown in unreplicated field plots at the experiment field in Maksimir, Zagreb (45.8293 N, 16.0334 E) in year 2014. They were classified as determinate (type I growth habit; Singh, 1982) or indeterminate. After preliminary analyses, accessions showing similar seed color/pattern and habit while collected from the same location (village) but from different households were excluded. Finally, 183 accessions were chosen and a single plant of each accession was used in the analyses. A list of the accessions, along with their “passport” information, as well as the information on habit, phaseolin type and cluster (based on model-based clustering methods, see below) is available in Table S1. Some examples of seed color/pattern diversity of accessions are shown in Figure S1. The accessions are held at the Department of Seed Science and Technology, Faculty of Agriculture, University of Zagreb.

### DNA extraction, phaseolin, and microsatellite analysis

Using Plant DNeasy 96 kit (Qiagen®), DNA was isolated from 25 mg of silica-gel dried leaves according to the manufacturer's instructions without any additional clean-up. Tailed PCR approach (Schuelke, 2000) was used for amplification of phaseolin sequences (Kami et al., 1995). The 20 μl of the PCR mix contained 2 pmol of the tailed forward primer (5′-TGTAAAACGACGGCCAGTAGCATATTCTAGAGGCCTCC-3′), 8 pmol of reverse (5′-GCTCAGTTCCTCAATCTGTTC-3′), 8 pmol of FAM labeled M13 primer (5′-TGTAAAACGACGGCCAGT-3), 1 × PCR buffer, 4 pmol of each dNTP, 0.5 U Taq™ HS DNA Polymerase (Takara Bio Inc.) and 5 ng of template DNA. PCR protocol with an initial touchdown cycles (94°C for 5 min; 5 cycles of 45 s at 94°C, 30 s at 60°C, which was lowered by 1°C in each cycle, and 90 s at 72°C; 25 cycles of 45 s at 94°C, 30 s at 55°C, and 90 s at 72°C; and 8-min extension step at 72°C) was employed (Radosavljević et al., 2011). The PCR products were sent to GeneScan service Macrogen® (South Korea) where they were detected on an ABI 3730xL DNA analyzer (Applied Biosystems®) by and analyzed GeneMapper 4.0 computer program (Applied Biosystems®).

Twenty-six PCR primer pairs were used for microsatellite analysis (Table S2). DNA amplification was performed using multiplex PCR mix and the same two-step PCR protocol with an initial touchdown cycle as in phaseolin type determination. The 20 μl of PCR mix contained 5 pmol of each of four fluorescent labeled forward primers (6-FAM, VIC, NED, PET; Applied Biosystems®), 5 pmol of reverse primers, 1 × PCR buffer, 4 pmol of each dNTP, 0.5 U Taq™ HS DNA Polymerase (Takara Bio Inc.) and 5 ng of template DNA. Fluorescent labeled PCR products were detected on an ABI 3730XL (Applied Biosystems®) by GeneScan service (Macrogen®). Allele sizes (in base pairs) of PCR products were estimated using GeneMapper 4.0 computer program (Applied Biosystems®).

### Data analysis

The average number of alleles per locus (*N*_*a*_), observed heterozygosity (*H*_*O*_), and gene diversity (expected heterozygosity; *H*_*E*_) for each microsatellite locus was calculated in GENEPOP 4.0 (Raymond and Rousset, 1995).

The proportion-of-shared-alleles distance (Bowcock et al., 1994) between pairs of accessions genotyped using 26 microsatellites was calculated using MICROSAT (Minch et al., 1997). Cluster analysis was performed using the Fitch-Margoliash least-squares algorithm in PHYLIP (Felsenstein, 2004). The reliability of the tree topology was assessed via bootstrapping (Felsenstein, 1985) over 1,000 replicates generated by MICROSAT and subsequently used in PHYLIP.

A model-based clustering method was applied on multilocus microsatellite data to infer genetic structure and define the number of clusters in the dataset using the software STRUCTURE ver. 2.3.3 (Pritchard et al., 2000). Thirty runs per each cluster (*K*) ranging from 1 to 11 were carried out on the Isabella computer cluster at the University of Zagreb, University Computing Centre (SRCE). Each run consisted of a burn-in period of 200,000 steps followed by 10^6^ MCMC (Monte Carlo Markov Chain) replicates assuming admixture model and correlated allele frequencies. No prior information was used to define the clusters. The choice of the most likely number of clusters (*K*) was carried out by comparing the average estimates of the likelihood of the data, *ln*[*Pr*(X|*K*)], for each value of *K* (Pritchard et al., 2000), as well as by calculating an ad hoc statistic Δ*K*, based on the rate of change in the log probability of data between successive *K*-values as described by Evanno et al. (2005). The program STRUCTURE HARVESTER v0.6.92 was used to process the STRUCTURE results files (Earl and von Holdt, 2012). Runs were clustered and averaged using CLUMPAK (Kopelman et al., 2015). The accessions were assigned to a particular cluster if an arbitrary value of *Q* > 75% of their genome was estimated to belong to that cluster (Matsuoka et al., 2002), while those accessions having the membership probabilities *Q* < 75% for each cluster were considered as of “mixed origin.” The likelihood-ratio chi-square test in SAS (SAS Institute, 2004) was used to test for dependence between phaseolin type and cluster membership of the accessions. The strength of association was assessed by calculating Cramér's V, the measure that reaches the maximum value of 1 when the two variables (i.e., classification criteria) are equal to each other.

Genetic diversity of Croatian common bean accessions was analyzed by classifying the accessions into two and into three germplasm groups according to the results of model-based cluster analyses of 26 microsatellite loci and phaseolin type. The accessions considered as of “mixed origin” as well as those that did not show correspondence between phasolin type and cluster membership in STRUCTURE were excluded from further analysis.

The genetic diversity of each group of accessions was assessed by calculating the average number of alleles per locus (*N*_*a*_) and allelic richness (*N*_*ar*_) in FSTAT (Goudet, 2002) as well as gene diversity (*H*_*E*_) in GENEPOP 4.0 (Raymond and Rousset, 1995). In order to compare the values of allelic richness (*N*_*ar*_) and gene diversity (*H*_*E*_) between/among groups, the repeated measures analysis of variance was carried out using PROC GLM in SAS followed by *post-hoc* Bonferroni's adjustments.

The analysis of molecular variance (AMOVA; Excoffier et al., 1992) using ARLEQUIN ver. 3.0 (Excoffier et al., 2005) was used to partition the total microsatellite diversity between and within groups of accessions. The variance components were tested statistically by non-parametric randomization tests using 10,000 permutations.

## Results

### Classification of croatian common bean accessions

Phaseolin analysis of 183 accessions revealed that 53 (28.96%) of them were of phaseolin type I (Mesoamerican; “S”), 42 (22.95%) of II (Andean; “H” or “C”) and 88 (48.09%) of III (Andean; “T”).

A total of 137 alleles were detected at 26 SSR loci, ranging from 2 (BMb469, BMd12, BMd22, BMd25, BMd45, BMd46, and BMd47) to 19 (PVat007) alleles per microsatellite locus with an average of 5.27 (Table S2). Observed heterozygosity (*H*_*O*_) values of all the markers were equal to zero, i.e., all the samples were completely homozygous for all the loci. Average gene diversity (*H*_*E*_) was *H*_*E*_ = 0.572, ranging from 0.389 (BMd12) to 0.885 (PVat007).

**Figure 2:**
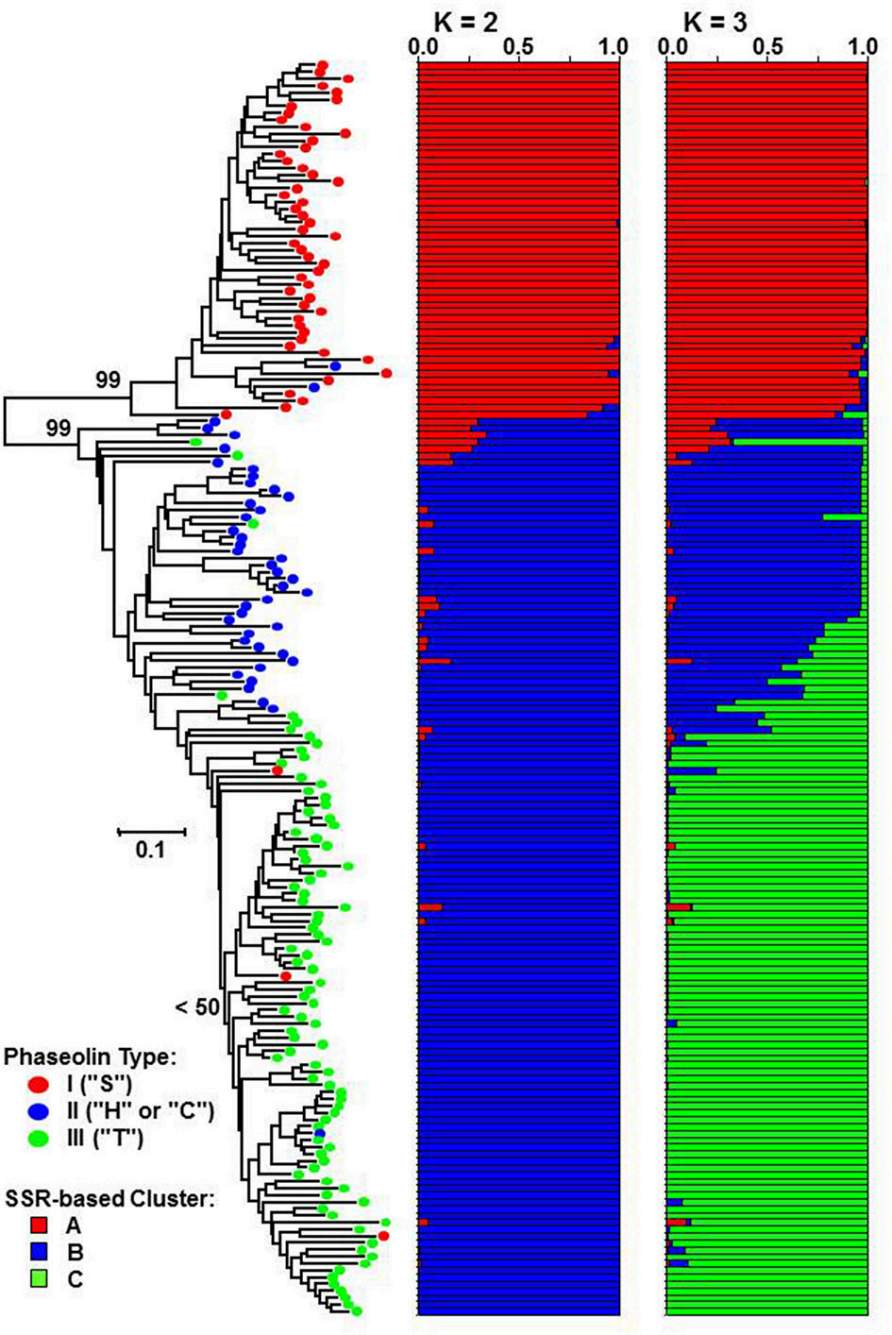
**Fitch-Margoliash tree based on proportion-of-shared alleles distance between 183 common bean accessions**. Average proportions of membership for *K* = 2 and 3 clusters are given as estimated by STRUCTURE. Classification based on phaseolin type is indicated on branches of the tree.

The genetic distance between pairs of accessions based on the proportion-of-shared-alleles distance measure ranged from *D*_*psa*_ = 0.038 (50 common alleles out of 52) to *D*_*psa*_ = 1.000 (no alleles in common) with the average of *D*_*psa*_ = 0.577. The Fitch-Margoliash tree grouped the accessions into two well-supported clades (bootstrap support value: 99%) corresponding to Mesoamerican and Andean origin of accessions as identified by phaseolin analysis (Figure 2). The subclade containing the great majority of phaseolin type III accessions could be identified within the Andean clade, although the monophyly of that group was not supported by a bootstrap value higher than 50%.

The STRUCTURE analysis, as expected, identified *K* = 2 as the most likely number of clusters (Δ*K* = 21410.10). The most of the accessions of Mesoamerican origin (phaseolin type I) were assigned to cluster A, while the accession of Andean origin (phaseolin type II and III) were assigned to cluster B. The association between phaseolin type and cluster membership was highly significant and nearly complete (χ^2^ = 174.72; *df* = 1; *P* < 0.0001; Cramér's *V* = 0.93). At *K* = 3, the newly formed cluster (cluster C) clearly grouped the phaseolin type III accessions that corresponded to the subclade in the Fitch-Margoliash tree (χ^2^ = 281.97; *df* = 4; *P* < 0.0001; Cramér's *V* = 0.89).

At *K* = 2, a total of five accessions could be considered as of “mixed origin” having the membership probabilities *Q* < 75% for both clusters. Furthermore, additional five accessions did not show the correspondence between phaseolin type and the membership according to model-based clustering analysis based on microsatellite loci (i.e., Mesoamerican group: cluster A/phaseolin type I; Andean group: cluster B phaseolin type II or III). Thus, a total of 10 accessions (5.46%) could be considered as putative hybrids between gene pools and were excluded from the subsequent analyses of genetic diversity.

At *K* = 3, a total of 16 accessions were classified as of “mixed origin,” while 13 accessions did not show the correspondence (i.e., Mesoamerican group: cluster A/phaseolin type I, Andean group B: cluster B/phaseolin type II; Andean group C: cluster C/phaseolin type III). As four accessions were classified as both of “mixed origin” and “non-corresponding,” a total of 25 accessions (13.66%) originating from hybridization among the three groups were excluded from the subsequent analyses of genetic diversity. Finally, the classification of 183 Croatian common bean accessions would the following: (1) 50 accessions (27.32%) belonged to Mesoamerican group, 27 (14.75%) to Andean group B and 81 (44.26%) to Andean group C; (2) Four (2.19%) accessions were putative hybrids between Mesoamerican group and Andean group B, another four (2.19%) were hybrids between Mesoamerican and Andean group C, while 17 (9.29) accessions were hybrids between Andean groups B and C.

### Genetic diversity of germplasm groups

By classifying accessions into two groups [Mesoamerican group: cluster A at *K* = 2/phaseolin type I vs. Andean group: cluster B at *K* = 2/phaseolin type II or III], the Andean group of accessions showed slightly higher values of allelic richness (*N*_*ar*_) as well as gene diversity (*H*_*E*_) than the Mesoamerican group but the differences were not significant following the analysis of variance (Table 1). Average genetic distance between pairs of accessions belonging to Mesoamerican group (*D*_*psa*_ = 0.277) was lower than in Andean group (*D*_*psa*_ = 0.356) while the average distance between pairs belonging to different groups was considerably higher (*D*_*psa*_ = 0.887). The analysis of molecular variance (AMOVA) revealed that that 63.34% of microsatellite diversity could be attributed to differences between groups (Φ_*ST*_ = 0.633; *P* < 0.0001; Table 2). Mesoamerican group of accessions consisted mostly of accessions of indeterminate growth habit (43 out of 50) while in the Andean group the accessions of determinate growth habit (85 out of 123). The association between group membership and growth habit was highly significant, but moderate (χ^2^ = 46.54; *df* = 1; *P* < 0.0001; Cramér's *V* = 0.50).

**Table 1:** **Genetic diversity of Croatian common bean accessions based on 26 microsatellite loci**.

**Cluster/Phaseolin type**	**N_Acc_**	**S**	**T**	***N_*a*_***	***N_*ar*_***	***N_*pr*_***	***H_*E*_***
A/I (Mesoamerican)	50	7	43	3.269	3.269	31	0.277
B/II or III (Andean)	123	85	38	4.038	3.677	51	0.356
*P*-value					0.455		0.249
A/I (Mesoamerican)	50	7	43	3.269	3.100	31	0.277
B/II (Andean)	27	0	27	2.885	2.885	12	0.314
C/III (Andean)	81	77	4	2.846	2.538	18	0.263
*P*-value					0.291		0.679

*Accessions were classified into two and three groups according to model-based cluster analysis and phaseolin type. N_Acc_, No. of Accessions; S, No. of Accessions with Determinate Growth Habit; T, No. of Accessions with Indeterminate Growth Habit; N_a_, No. of Alleles; N_ar_, Allelic Richness; N_pr_, No. of Private Alleles; H_E_, Gene Diversity; P-value, significance level of the F-test*.

**Table 2:** **Analysis of molecular variance for the partitioning of microsatellite diversity of Croatian common bean accessions classified into (A) two as well as (B) three groups according to model-based cluster analysis and phaseolin type**.

**Analysis**	**Source of variation**	**df**	**Variance components**	**% Total variance**	**Φ-statistic**	***P(Φ)***
(A)	Between groups	1	7.443	63.34	0.633	<0.0001
	Within groups	344	4.308	36.66		
(B)	Among groups	2	6.433	64.44	0.644	<0.0001
	Within groups	313	3.550	35.56		

*P(Φ)–Φ -statistic probability level after 10,000 permutations*.

The diversity analysis of three groups (Mesoamerican group: cluster A at K = 3/phaseolin type I, Andean group B: cluster B at *K* = 3/phaseolin type II; Andean group C: cluster C at *K* = 3/phaseolin type III) revealed that the Mesoamerican group had the highest allelic richness (*N*_*ar*_) while the Andean group B had the highest gene diversity (*H*_*E*_). The lowest values of both measures were found in the Andean group C. However, the differences among groups were not significant (Table 1). The AMOVA analysis based on three groups revealed the similar results as in case of classification into two groups: 64.44% of diversity was attributed to differences between groups (Φ_*ST*_ = 0.644; *P* < 0.0001; Table 2). The lowest pairwise Φ_*ST*_ value between groups types was found between two Andean groups (Φ_*ST*(*B*/*C*)_ = 0.420) while the Φ_*ST*_-values between Mesoamerican group and Andean group B as well as between Mesoamerican group and Andean group C were considerably higher (Φ_*ST*(*A*/*B*)_ = 0.654; Φ_*ST*/(*A*/*C*)_ = 0.706). All the pairwise Φ_*ST*_-values were highly significant (*P* < 0.0001). The same pattern was found by analyzing the average genetic distance between pairs of accessions belonging to different groups. The average distance between Andean groups A and B (*D*_*psa*(*B*/*C*)_ = 0.481) was substantially lower than the distances between Mesoamerican group and Andean groups (*D*_*psa*(*A*/*B*)_ = 0.831; *D*_*psa*(*A*/*C*)_ = 0.908). As already mentioned, the Mesoamerican group of accessions consisted mostly of accessions of indeterminate growth habit (43 out of 50). Moreover, the Andean group B included exclusively the accession of indeterminate growth habit, while the great majority of accessions belonging to Andean group C was of determinate growth habit (77 out of 81) leading to a strong association between group membership and growth habit (χ^2^ = 146.04; *df* = 2; *P* < 0.0001; Cramér's *V* = 0.87). Thus, the estimated probability of correct prediction of growth habit based on phaseolin type of accession was *P* = 0.93.

## Discussion

### Origin of croatian common bean germplasm

To determine the evolutionary origin of Croatian common bean accessions we combined the results of phaseolin marker analysis and microsatellite genotyping. Out of 183 accessions, 27.32% are of Mesoamerican origin, 68.31% of Andean, while 4.37% of accessions represent putative hybrids between gene pools. Our results are in line with the findings of numerous previous studies that the European common bean germplasm originates from both gene pools, Mesoamerican and Andean, later being more frequently found (see Bellucci et al., 2014 for a review). The proportion of landraces of the Mesoamerican origin tends to increase in eastern and south-eastern Europe as shown, e.g., in case of Albania (Logozzo et al., 2007), Bulgaria (Svetleva et al., 2006), Macedonia (Maras et al., 2015) and Greece (Lioi, 1989). However, Maras et al. (2015) reported that the proportions found in accessions from Bosnia and Herzegovina, Croatia, Serbia and Slovenia were very similar to those found in the Iberian Peninsula and Italy indicating that common bean was introduced into the western Balkans mainly from the Mediterranean Basin. In case of Croatia these findings have been confirmed by the present study that included substantially more accessions. However, in contrast to Italian and Spanish common bean germplasm in which the Andean phaseolin type is “C” prevails over the type “T” (Logozzo et al., 2007; Angioi et al., 2010; Raggi et al., 2013), “T” (i.e., Andean group C: cluster C at *K* = 3/phaseolin type III) is clearly the most common phaseolin type found in Croatian germplasm as in most other European countries (Logozzo et al., 2007).

### Genetic diversity of the mesoamerican and andean group of accessions

Nearly complete correspondence of classifications based on phaseolin analysis and model-based clustering using microsatellite markers as well as a strong association between group membership and growth habit in Croatian common bean germplasm could be explained by a series of sequential bottlenecks during domestication, early introduction to Portugal and Spain and eastward expansion throughout Europe. The Mesoamerican group of accessions as well as the Andean group B consists mostly of accessions of indeterminate growth habit while the great majority of accessions belonging to Andean group C have determinate growth habit. A strong association between phaseolin pattern and growth habit was reported by Raggi et al. (2013) by analyzing Italian common bean landraces: plants with climbing ability were prevalent in the “C” (Mesoamerican group) and “S” (Andean group B) phaseolin pattern groups, while bush plants were prevalent in the “T” (Andean group C) group. Moreover, Kwak et al. (2012) reported that determinate types were found mainly in Andean subpopulations.

Similar levels of gene diversity of Croatian common bean accessions of Andean origin as compared with those of Mesoamerican origin are in line with findings previously reported by Santalla et al. (2002) in Iberian landraces as well as by Angioi et al. (2010) at European level, while in the domestication centers, the diversity observed in Mesoamerican gene pool is higher than in the Andean (Kwak and Gepts, 2009). Angioi et al. (2010) offered two plausible, and not mutually exclusive, explanations: (a) further selection in Europe might have reduced the variation of the Mesoamerican germplasm, and/or (b) diversity of Mesoamerican introductions to Europe was already reduced when compared with the Mesoamerican gene pool. Additionally, the apparent incongruence can be explained by the fact that the Andean gene pool is represented by two separate groups of accessions (determinate/indeterminate) that resulted from divergent selection during domestication in the Andes. It is well-documented that the divergence between Mesoamerican and Andean gene pools preceded the domestication that occurred independently in two geographic regions (Kwak and Gepts, 2009). The wild Andean gene pool diverged from the wild Mesoamerican gene pool, the origin of the species (Bitocchi et al., 2012), with a strong bottleneck, as shown by considerably lower genetic diversity of wild Andean gene pool in comparison to Mesoamerican (Schmutz et al., 2014). Wild common beans are all indeterminate and it would be reasonable to assume that the first domesticated types were also indeterminate as determinacy is a trait selected during or after domestication (Kwak et al., 2012). In Mesoamerican domestication center, indeterminate types were the valuable component of traditional maize-bean-squash multicrop system (Zizumbo-Villarreal and Colunga-García, 2010). On the other hand, in Andean domestication center, early farmers did not have a suitable crop that could serve as a physical support for viny common bean as domestication of common bean preceded the introduction of maize (Kwak et al., 2012). By constant selection of genotypes displaying a more compact growth habit, determinacy was included in the group of traits known as the domestication syndrome (Hammer, 1984; Koinange et al., 1996). Indeterminate landraces were not abandoned, and after maize introduction, maize/bean intercropping has gained importance and remained until nowadays (Lithourgidis et al., 2011). Although the majority of determinate type accessions originate from the Andean gene pool, the determinacy has been selected independently in both domestication centers (Kwak et al., 2012). The process of domestication led to reduction of genetic diversity, but, interestingly, the bottleneck effect was threefold greater in Mesoamerica as compared to the Andes as shown by Bitocchi et al. (2013). Bearing in mind that the wild Andean gene pool was strongly impoverished as a result of a bottleneck that occurred before domestication, Bitocchi et al. (2013) concluded that it would be the reason why the subsequent domestication bottleneck had the minor effect in the Andes. Another possible explanation would be that the divergent selection (indeterminate/determinate types) during domestication that was prominent in the Andes but presumably negligible in the Mesoamerican region. While in Mesoamerica the reduction in diversity of neutral genes followed the reduction of the genes under selection, divergent selection in the Andes maintained or even increased diversity by possible effects of introgression/interchange during improvement (Burger et al., 2008).

### Hybridization between mesoamerican and andean gene pools

Out of 183 accessions, a total of 25 (13.66%) were classified as putative hybrids: Four (2.19%) accessions were putative hybrids between Mesoamerican group and Andean group B, another four (2.19%) were hybrids between Mesoamerican and Andean group C, while 17 (9.29%) accessions were hybrids between Andean groups B and C. Thus, the proportion of hybrids between Mesoamerican and Andean gene pools amounts to only 4.37%. This result is in striking disagreement with the results reported by, *inter alia*, Angioi et al. (2010) and Gioia et al. (2013). By analyzing 307 European common bean accessions they estimated that about 44% of them were derived from hybridization between Mesoamerican and Andean gene pools. Similarly, Gioia et al. (2013) reported that 40.2% of 256 European common bean accessions derived from hybridization between two gene pools. Moreover, Angioi et al. (2010) showed that the hybridization was particularly frequent in Central Europe as compared to Italy and the Iberian Peninsula. Our results show a very different picture with much less frequent hybridization compared to Angioi et al. (2010) and Gioia et al. (2013). Thus, in Croatian common bean landraces inter-gene pool hybridization appears to have been very limited, even if differences in the methods used to detect the hybrids should also be considered when comparing our results to that obtained in the rest of Europe. Indeed, Angioi et al. (2010) carried out the analysis by combining chloroplast microsatellites and two nuclear loci (for phaseolin types and *Pv-shatterproof1*), and Gioia et al. (2013) used nuclear and chloroplast microsatellites as well as two nuclear loci (for phaseolin types and *Pv-shatterproof1*), while this study was based on nuclear microsatellites and phaseolin marker.

## Conclusion

This study provides a comprehensive picture of genetic diversity and structure of Croatian common bean germplasm. Out of 183 accessions, 27.32% were of Mesoamerican origin, 68.31% of Andean, while 4.37% of accessions represented putative hybrids between gene pools. For the most part, the classification of common bean accessions according to phaseolin type analysis was in congruence with the results of both distance-based and model-based analyses of microsatellite marker data. The Mesoamerican group (cluster A/phaseolin type I) of accessions as well as the Andean group B (cluster B/phaseolin type II) consisted mostly of accessions of indeterminate growth habit while the great majority of accessions belonging to Andean group C (cluster C/phaseolin type III) had determinate growth habit. Nearly complete correspondence of classifications based on phaseolin analysis and microsatellite markers as well as a strong association between group membership and growth habit in Croatian common bean germplasm could be explained by a series of sequential bottlenecks.

## Author contributions

Conceived and designed the manuscript: KC, MV, and ZŠ. Contributed to analysis: ZL, MV, AB, and MG. Analyzed the data: ZL and ZŠ. Wrote the manuscript: KC, ZL, MV, AB, MG, BL, and ZŠ.

### Conflict of interest statement

The authors declare that the research was conducted in the absence of any commercial or financial relationships that could be construed as a potential conflict of interest.
