# Supplementary material for: Genetic Diversity of Croatian Common Bean Landraces
Source: Front Plant Sci. 2017 Apr 20;8:604. doi: 10.3389/fpls.2017.00604 (PMC5397504; doi:10.3389/fpls.2017.00604)
Supplement: Supplementary file 1 [file Table1.DOC]

**Table S1 –** Information of the common bean accessions used in this study.

| **Accession** | **Region** | **County** | **Location** | **Latitude** | **Longitude** | **Altitude (m a.s.l.)** | **Habit** | **Phaseolin type** | **Cluster membership (K = 3)** |
| --- | --- | --- | --- | --- | --- | --- | --- | --- | --- |
| Ph001 | Pannonian | Vukovar-Srijem | Tovarnik | 45.16N | 19.15E | 89 | D | III | C |
| Ph002 | Pannonian | Vukovar-Srijem | Kusać-Soljani | 44.95N | 18.97E | 82 | D | III | C |
| Ph007 | Pannonian | Osijek-Baranja | Podravska Moslavina | 45.78N | 17.98E | 100 | D | III | C |
| Ph008 | Pannonian | Osijek-Baranja | Podravska Moslavina | 45.78N | 17.98E | 100 | I | I | A |
| Ph009 | Pannonian | Zagreb | Cvetković | 45.65N | 15.65E | 125 | D | I | A |
| Ph010 | Pannonian | Zagreb | Cvetković | 45.65N | 15.65E | 125 | D | III | C |
| Ph011 | Pannonian | Zagreb | Cvetković | 45.65N | 15.65E | 125 | I | I | A |
| Ph013 | Pannonian | Zagreb | Cvetković | 45.65N | 15.65E | 125 | I | II | B |
| Ph014 | Pannonian | Zagreb | Cvetković | 45.65N | 15.65E | 125 | I | I | A |
| Ph015 | Pannonian | Zagreb | Cvetković | 45.65N | 15.65E | 125 | D | III | C |
| Ph016 | Pannonian | Zagreb | Cvetković | 45.65N | 15.65E | 125 | I | II | B |
| Ph017 | Pannonian | Zagreb | Cvetković | 45.65N | 15.65E | 125 | D | III | C |
| Ph019 | Pannonian | Varaždin | Grešćevina | 46.23N | 16.47E | 186 | I | I | A |
| Ph020 | Pannonian | Varaždin | Grešćevina | 46.23N | 16.47E | 186 | D | III | C |
| Ph021 | Pannonian | Bjelovar-Bilogora | Turčević polje | 45.71N | 17.33E | 200 | D | III | C |
| Ph022 | Pannonian | Bjelovar-Bilogora | Turčević polje | 45.71N | 17.33E | 200 | I | I | A |
| Ph023 | Pannonian | Zagreb | Cvetković | 45.65N | 15.65E | 125 | I | I | A |
| Ph025 | Pannonian | Zagreb | Cvetković | 45.65N | 15.65E | 125 | D | II | B |
| Ph031 | Mediterranean | Dubrovnik-Neretva | Mlinište | 42.99N | 17.62E | 10 | D | III | C |
| Ph032 | Pannonian | Varaždin | Mali Bukovec | 46.29N | 16.74E | 140 | D | I | A |
| Ph037 | Pannonian | Sisak-Moslavina | Staro Pračno | 45.49N | 16.35E | 98 | I | I | A |
| Ph042 | Pannonian | Međimurje | Ivanovec | 46.37N | 16.48E | 160 | I | I | A |
| Ph044 | Pannonian | Međimurje | Ivanovec | 46.37N | 16.48E | 160 | D | I | A |
| Ph045 | Pannonian | Međimurje | Ivanovec | 46.37N | 16.48E | 160 | D | III | C |
| Ph046 | Pannonian | Međimurje | Ivanovec | 46.37N | 16.48E | 160 | I | I | A |
| Ph049 | Pannonian | Međimurje | Gornji Hrašćan | 46.38N | 16.34E | 172 | D | III | C |
| Ph052 | Pannonian | Međimurje | Gornji Hrašćan | 46.38N | 16.34E | 172 | D | III | C |
| Ph053 | Pannonian | Međimurje | Slakovec | 46.41N | 16.37E | 183 | I | I | A |
| Ph055 | Pannonian | Međimurje | Šandorovec | 46.33N | 16.42E | 165 | D | III | C |
| Ph063 | Pannonian | Međimurje | Novo Selo Rok | 46.43N | 16.46E | 195 | D | III | C |
| Ph066 | Pannonian | Međimurje | Novo Selo Rok | 46.43N | 16.46E | 195 | D | III | C |
| Ph070 | Pannonian | Zagreb | Sveti Ivan Zelina | 45.96N | 16.25E | 171 | D | III | C |
| Ph071 | Pannonian | Zagreb | Sveti Ivan Zelina | 45.96N | 16.25E | 171 | D | III | C |
| Ph074 | Pannonian | Međimurje | Sveti Martin na Muri | 46.53N | 16.36E | 170 | D | III | C |
| Ph078 | Pannonian | Međimurje | Slakovec | 46.41N | 16.37E | 183 | D | III | C |
| Ph079 | Pannonian | Međimurje | Dragoslavec | 46.45N | 16.35E | 320 | I | II | B |
| Ph080 | Pannonian | Međimurje | Dragoslavec | 46.45N | 16.35E | 320 | D | III | C |
| Ph084 | Pannonian | Varaždin | Oštrice | 46.19N | 16.35E | 191 | D | III | C |
| Ph085 | Pannonian | Varaždin | Oštrice | 46.19N | 16.35E | 191 | D | III | C |
| Ph088 | Pannonian | Varaždin | Presečno | 46.09N | 16.35E | 220 | D | III | C |
| Ph089 | Pannonian | Varaždin | Oštrice | 46.19N | 16.35E | 191 | I | II | B |
| Ph092 | Pannonian | Varaždin | Oštrice | 46.19N | 16.35E | 191 | D | III | C |
| Ph094 | Pannonian | Varaždin | Oštrice | 46.19N | 16.35E | 191 | D | III | C |
| Ph099 | Pannonian | Varaždin | Remetinec | 46.18N | 16.32E | 234 | D | III | C |
| Ph106 | Pannonian | Krapina-Zagorje | Gredice | 46.03N | 15.73E | 180 | I | I | A |
| Ph107 | Pannonian | Krapina-Zagorje | Gredice | 46.03N | 15.73E | 180 | I | II | B |
| Ph108 | Pannonian | Krapina-Zagorje | Lučelnica | 45.60N | 15.92E | 130 | I | II | B |
| Ph110 | Pannonian | Krapina-Zagorje | Lučelnica | 45.60N | 15.92E | 130 | I | II | B |
| Ph112 | Pannonian | Krapina-Zagorje | Lučelnica | 45.60N | 15.92E | 130 | I | II | B |
| Ph115 | Pannonian | Krapina-Zagorje | Lučelnica | 45.60N | 15.92E | 130 | I | II | B |
| Ph116 | Pannonian | Krapina-Zagorje | Lučelnica | 45.60N | 15.92E | 130 | I | II | B |
| Ph121 | Pannonian | Krapina-Zagorje | Lučelnica | 45.60N | 15.92E | 130 | I | II | B |
| Ph122 | Pannonian | Krapina-Zagorje | Lučelnica | 45.60N | 15.92E | 130 | I | II | B |
| Ph124 | Pannonian | Krapina-Zagorje | Lučelnica | 45.60N | 15.92E | 130 | I | I | A |
| Ph125 | Pannonian | Krapina-Zagorje | Tomaševec | 46.03N | 15.77E | 170 | I | II | B |
| Ph126 | Pannonian | Krapina-Zagorje | Dol Klanječki | 46.05N | 15.75E | 231 | I | II | B |
| Ph130 | Pannonian | Krapina-Zagorje | Dol Klanječki | 46.05N | 15.75E | 231 | I | I | A |
| Ph134 | Pannonian | Krapina-Zagorje | Dol Klanječki | 46.05N | 15.75E | 231 | I | II | B |
| Ph137 | Pannonian | Krapina-Zagorje | Lučelnica | 45.60N | 15.92E | 130 | I | I | A |
| Ph138 | Pannonian | Krapina-Zagorje | Tomaševec | 46.03N | 15.77E | 170 | I | II | B |
| Ph139 | Pannonian | Krapina-Zagorje | Lučelnica | 45.60N | 15.92E | 130 | D | III | C |
| Ph140 | Pannonian | Krapina-Zagorje | Lučelnica | 45.60N | 15.92E | 130 | D | III | C |
| Ph152 | Pannonian | Varaždin | Mali Bukovec | 46.29N | 16.74E | 140 | I | II | B |
| Ph156 | Pannonian | Krapina-Zagorje | Dol Klanječki | 46.05N | 15.75E | 231 | I | I | A |
| Ph162 | Pannonian | Krapina-Zagorje | Dol Klanječki | 46.05N | 15.75E | 231 | I | II | B |
| Ph166 | Pannonian | Varaždin | Remetinec | 46.18N | 16.32E | 234 | I | I | A |
| Ph167 | Pannonian | Varaždin | Remetinec | 46.18N | 16.32E | 234 | I | II | B |
| Ph169 | Pannonian | Varaždin | Visočka | 46.17N | 16.39E | 189 | D | III | C |
| Ph170 | Pannonian | Varaždin | Visočka | 46.17N | 16.39E | 189 | D | III | C |
| Ph176 | Pannonian | Zagreb | Dubrava | 45.84N | 16.54E | 140 | D | III | C |
| Ph178 | Pannonian | Zagreb | Dubrava | 45.84N | 16.54E | 140 | I | I | A |
| Ph180 | Pannonian | Zagreb | Dubrava | 45.84N | 16.54E | 140 | D | III | C |
| Ph184 | Pannonian | Zagreb | Dubrava | 45.84N | 16.54E | 140 | I | II | B |
| Ph185 | Pannonian | Međimurje | Totovec | 46.34N | 16.44E | 163 | I | III | C |
| Ph189 | Pannonian | Međimurje | Totovec | 46.34N | 16.44E | 163 | I | II | B |
| Ph190 | Pannonian | Međimurje | Totovec | 46.34N | 16.44E | 163 | I | II | B |
| Ph191 | Pannonian | Međimurje | Totovec | 46.34N | 16.44E | 163 | I | I | A |
| Ph192 | Pannonian | Varaždin | Leštakovec | 46.25N | 16.45E | 180 | I | I | A |
| Ph195 | Pannonian | Varaždin | Leštakovec | 46.25N | 16.45E | 180 | D | III | C |
| Ph196 | Pannonian | Osijek-Baranja | Tvrđavica | 45.57N | 18.68E | 87 | D | III | C |
| Ph197 | Pannonian | Osijek-Baranja | Bijelo brdo | 45.52N | 18.87E | 94 | D | III | C |
| Ph198 | Pannonian | Osijek-Baranja | Ivanovac | 45.49N | 18.64E | 85 | D | III | C |
| Ph199 | Pannonian | Vukovar-Srijem | Tordinci | 45.37N | 18.79E | 86 | D | III | C |
| Ph200 | Pannonian | Osijek-Baranja | Lug | 45.66N | 18.77E | 90 | D | III | C |
| Ph203 | Pannonian | Krapina-Zagorje | Lučelnica | 45.60N | 15.92E | 130 | D | III | C |
| Ph205 | Pannonian | Osijek-Baranja | Ernestinovo | 45.45N | 18.66E | 85 | D | I | A |
| Ph206 | Pannonian | Osijek-Baranja | Ernestinovo | 45.45N | 18.66E | 85 | D | III | C |
| Ph207 | Mountainous | Primorje-Gorski Kotar | Delnice | 45.40N | 14.80E | 706 | I | III | C |
| Ph208 | Mountainous | Primorje-Gorski Kotar | Delnice | 45.40N | 14.80E | 706 | I | III | C |
| Ph209 | Mountainous | Primorje-Gorski Kotar | Delnice | 45.40N | 14.80E | 706 | D | III | C |
| Ph211 | Mountainous | Primorje-Gorski Kotar | Lokve | 45.36N | 14.75E | 726 | D | III | C |
| Ph215 | Pannonian | Bjelovar-Bilogora | Plošćica | 45.73N | 16.92E | 130 | I | I | A |
| Ph216 | Pannonian | Bjelovar-Bilogora | Plošćica | 45.73N | 16.92E | 130 | D | III | C |
| Ph218 | Mountainous | Primorje-Gorski Kotar | Prezid | 45.64N | 14.58E | 770 | I | I | A |
| Ph220 | Mountainous | Primorje-Gorski Kotar | Prezid | 45.64N | 14.58E | 770 | D | I | A |
| Ph222 | Mountainous | Primorje-Gorski Kotar | Prezid | 45.64N | 14.58E | 770 | D | III | C |
| Ph223 | Mountainous | Primorje-Gorski Kotar | Prezid | 45.64N | 14.58E | 770 | D | III | C |
| Ph224 | Mountainous | Primorje-Gorski Kotar | Prezid | 45.64N | 14.58E | 770 | I | II | B |
| Ph230 | Pannonian | Karlovac | Orlovac | 45.53N | 15.56E | 110 | D | III | C |
| Ph232 | Pannonian | Karlovac | Orlovac | 45.53N | 15.56E | 110 | D | III | C |
| Ph233 | Pannonian | Varaždin | Veliki Bukovec | 46.29N | 16.71E | 141 | I | II | B |
| Ph235 | Pannonian | Varaždin | Veliki Bukovec | 46.29N | 16.71E | 141 | I | I | A |
| Ph236 | Pannonian | Koprivnica-Križevci | Gregurovec | 46.16N | 15.98E | 230 | D | III | C |
| Ph239 | Pannonian | Zagreb | Rakitovec | 45.66N | 16.14E | 101 | D | III | C |
| Ph240 | Pannonian | Zagreb | Rakitovec | 45.66N | 16.14E | 101 | D | III | C |
| Ph241 | Pannonian | Zagreb | Rakitovec | 45.66N | 16.14E | 101 | I | II | B |
| Ph243 | Pannonian | Koprivnica-Križevci | Gračina | 45.98N | 16.56E | 160 | D | III | C |
| Ph245 | Pannonian | Koprivnica-Križevci | Gračina | 45.98N | 16.56E | 160 | I | II | B |
| Ph246 | Pannonian | Koprivnica-Križevci | Gračina | 45.98N | 16.56E | 160 | I | I | A |
| Ph247 | Pannonian | Koprivnica-Križevci | Gračina | 45.98N | 16.56E | 160 | I | I | A |
| Ph248 | Pannonian | Koprivnica-Križevci | Gračina | 45.98N | 16.56E | 160 | D | III | C |
| Ph249 | Pannonian | Koprivnica-Križevci | Gračina | 45.98N | 16.56E | 160 | D | III | C |
| Ph253 | Pannonian | Koprivnica-Križevci | Kloštar Podravski | 45.98N | 17.16E | 118 | D | III | C |
| Ph255 | Pannonian | Koprivnica-Križevci | Kloštar Podravski | 45.98N | 17.16E | 118 | D | II | B |
| Ph258 | Pannonian | Koprivnica-Križevci | Donji Fodrovec | 46.04N | 16.41E | 142 | D | II | B |
| Ph259 | Pannonian | Zagreb | Brčevec | 45.87N | 16.41E | 110 | D | III | C |
| Ph263 | Pannonian | Međimurje | Podturen | 46.47N | 16.55E | 155 | D | I | A |
| Ph265 | Pannonian | Međimurje | Sveta Marija | 46.33N | 16.74E | 140 | I | I | A |
| Ph268 | Pannonian | Međimurje | Podturen | 46.47N | 16.55E | 155 | I | I | A |
| Ph271 | Pannonian | Koprivnica-Križevci | Koprivnica | 46.16N | 16.83E | 135 | I | I | A |
| Ph278 | Pannonian | Koprivnica-Križevci | Peteranec | 46.19N | 16.89E | 130 | I | I | A |
| Ph280 | Pannonian | Krapina-Zagorje | Ladislavec | 46.11N | 16.06E | 200 | D | III | C |
| Ph282 | Pannonian | Krapina-Zagorje | Ladislavec | 46.11N | 16.06E | 200 | D | III | C |
| Ph287 | Pannonian | Koprivnica-Križevci | Zamladinec | 46.08N | 16.49E | 160 | D | III | C |
| Ph288 | Pannonian | Koprivnica-Križevci | Podgajec | 46.03N | 16.49E | 190 | I | I | A |
| Ph289 | Pannonian | Koprivnica-Križevci | Koprivnica | 46.16N | 16.83E | 135 | D | II | B |
| Ph293 | Pannonian | Koprivnica-Križevci | Selanec | 46.08N | 16.48E | 175 | D | I | A |
| Ph294 | Pannonian | Koprivnica-Križevci | Selanec | 46.08N | 16.48E | 175 | D | III | C |
| Ph295 | Pannonian | Koprivnica-Križevci | Selanec | 46.08N | 16.48E | 175 | I | II | B |
| Ph298 | Pannonian | Koprivnica-Križevci | Poljana Križevačka | 45.97N | 16.54E | 130 | D | III | C |
| Ph299 | Pannonian | Koprivnica-Križevci | Dijankovec | 46.04N | 16.48E | 174 | I | II | B |
| Ph300 | Pannonian | Koprivnica-Križevci | Dijankovec | 46.04N | 16.48E | 174 | I | II | B |
| Ph302 | Pannonian | Koprivnica-Križevci | Dijankovec | 46.04N | 16.48E | 174 | I | I | A |
| Ph303 | Pannonian | Bjelovar-Bilogora | Bjelovar | 45.90N | 16.84E | 132 | D | III | C |
| Ph304 | Pannonian | Bjelovar-Bilogora | Bjelovar | 45.90N | 16.84E | 132 | D | III | C |
| Ph305 | Pannonian | Koprivnica-Križevci | Gornji Dubovec | 46.01N | 16.44E | 136 | I | I | A |
| Ph306 | Pannonian | Koprivnica-Križevci | Gornji Dubovec | 46.01N | 16.44E | 136 | D | III | C |
| Ph307 | Pannonian | Koprivnica-Križevci | Gornji Dubovec | 46.01N | 16.44E | 136 | D | I | A |
| Ph308 | Pannonian | Koprivnica-Križevci | Gornji Dubovec | 46.01N | 16.44E | 136 | D | III | C |
| Ph310 | Pannonian | Koprivnica-Križevci | Greberanec | 45.92N | 16.37E | 175 | D | III | C |
| Ph312 | Pannonian | Koprivnica-Križevci | Greberanec | 45.92N | 16.37E | 175 | D | III | C |
| Ph314 | Pannonian | Koprivnica-Križevci | Dijankovec | 46.04N | 16.48E | 174 | D | III | C |
| Ph315 | Pannonian | Koprivnica-Križevci | Dijankovec | 46.04N | 16.48E | 174 | D | III | C |
| Ph317 | Pannonian | Koprivnica-Križevci | Podgajec | 46.03N | 16.49E | 190 | D | III | C |
| Ph318 | Pannonian | Koprivnica-Križevci | Kloštar | 46.01N | 17.21E | 110 | I | I | A |
| Ph319 | Pannonian | Koprivnica-Križevci | Podgajec | 46.03N | 16.49E | 190 | I | I | A |
| Ph320 | Pannonian | Koprivnica-Križevci | Zamladinec | 46.08N | 16.49E | 160 | D | I | A |
| Ph331 | Pannonian | Koprivnica-Križevci | Torčec | 46.22N | 16.89E | 128 | I | III | C |
| Ph334 | Pannonian | Vukovar-Srijem | Stari Mikanovci | 45.28N | 18.56E | 91 | D | III | C |
| Ph335 | Pannonian | Međimurje | Donji Pustakovec | 46.38N | 16.60E | 150 | D | II | B |
| Ph337 | Pannonian | Krapina-Zagorje | Marinci | 46.13N | 15.73E | 174 | I | II | B |
| Ph339 | Pannonian | Međimurje | Ivanovec | 46.37N | 16.48E | 160 | I | I | A |
| Ph343 | Pannonian | Karlovac | Vojnić | 45.33N | 15.70E | 140 | D | III | C |
| Ph344 | Mountainous | Lika-Senj | Brinje | 45.00N | 15.13E | 471 | D | III | C |
| Ph345 | Mountainous | Lika-Senj | Brinje | 45.00N | 15.13E | 471 | I | I | A |
| Ph346 | Mountainous | Lika-Senj | Brinje | 45.00N | 15.13E | 471 | D | III | C |
| Ph351 | Mountainous | Lika-Senj | Brinje | 45.00N | 15.13E | 471 | D | III | C |
| Ph352 | Pannonian | Virovitica-Podravina | Virovitica | 45.83N | 17.39E | 120 | I | I | A |
| Ph357 | Pannonian | Sisak-Moslavina | Šatornja | 45.34N | 16.01E | 150 | D | III | C |
| Ph358 | Mountainous | Karlovac | Ogulin | 45.27N | 15.22E | 320 | I | III | C |
| Ph361 | Mediterranean | Zadar | Škabrnja | 44.09N | 15.45E | 105 | D | III | C |
| Ph362 | Mediterranean | Zadar | Lišane ostrovičke | 43.97N | 15.76E | 133 | D | III | C |
| Ph363 | Pannonian | Virovitica-Podravina | Virovitica | 45.83N | 17.39E | 120 | D | III | C |
| Ph365 | Pannonian | Bjelovar-Bilogora | Plošćica | 45.73N | 16.92E | 130 | D | III | C |
| Ph366 | Pannonian | Bjelovar-Bilogora | Plošćica | 45.73N | 16.92E | 130 | I | I | A |
| Ph370 | Mountainous | Lika-Senj | Gospić | 44.55N | 15.37E | 561 | I | II | B |
| Ph371 | Mountainous | Lika-Senj | Gospić | 44.55N | 15.37E | 561 | D | III | C |
| Ph378 | Pannonian | Krapina-Zagorje | Brezakovec | 46.10N | 15.65E | 263 | I | I | A |
| Ph384 | Mediterranean | Šibenik-Knin | Piramatovci | 43.91N | 15.83E | 180 | D | III | C |
| Ph386 | Mediterranean | Šibenik-Knin | Piramatovci | 43.91N | 15.83E | 180 | I | I | A |
| Ph387 | Pannonian | Virovitica-Podravina | Pitomača | 45.95N | 17.23E | 116 | I | I | A |
| Ph388 | Pannonian | Virovitica-Podravina | Pitomača | 45.95N | 17.23E | 116 | I | II | B |
| Ph389 | Pannonian | Virovitica-Podravina | Pitomača | 45.95N | 17.23E | 116 | I | III | C |
| Ph390 | Pannonian | Virovitica-Podravina | Pitomača | 45.95N | 17.23E | 116 | I | II | B |
| Ph392 | Pannonian | Koprivnica-Križevci | Severovci | 46.07N | 17.11E | 114 | I | II | B |
| Ph393 | Pannonian | Koprivnica-Križevci | Severovci | 46.07N | 17.11E | 114 | I | II | B |
| Ph394 | Pannonian | Koprivnica-Križevci | Severovci | 46.07N | 17.11E | 114 | I | II | B |
| Ph395 | Pannonian | Virovitica-Podravina | Pitomača | 45.95N | 17.23E | 116 | D | III | C |
| Ph400 | Pannonian | Koprivnica-Križevci | Ferdinandovac | 46.06N | 17.19E | 111 | I | II | B |
| Ph406 | Pannonian | Koprivnica-Križevci | Podravske Sesvete | 46.00N | 17.21E | 110 | I | I | A |
| Ph409 | Pannonian | Brod-Posavina | Davor | 45.11N | 17.51E | 90 | I | I | A |
| Ph411 | Pannonian | Požega-Slavonia | Sesvete | 45.34N | 17.83E | 170 | I | I | A |
| Ph413 | Pannonian | Požega-Slavonia | Sesvete | 45.34N | 17.83E | 170 | I | I | A |
