# Supplementary material for: Genetic Diversity of Croatian Common Bean Landraces
Source: Front Plant Sci. 2017 Apr 20;8:604. doi: 10.3389/fpls.2017.00604 (PMC5397504; doi:10.3389/fpls.2017.00604)
Supplement: Supplementary file 2 [file Table2.DOC]

Table S2. Microsatellite markers used in the study

| Marker | Forward and reverse PCR primers | Repeat Motif | N_a_ | H*_E_* | Reference |
| --- | --- | --- | --- | --- | --- |
| BM143 | gggaaatgaacagaggaaa  atgttgggaacttttagtgtg | (GA)_35_ | 12 | 0,837 | a |
| BM151 | cacaacaagaaagacctcct  ttatgtattagaccacattacttcc | (CT)_14_ | 5 | 0,641 | a |
| BM157 | acttaacaaggaatagccacaca  gttaattgtttccaatatcaacctg | (GA)_16_ | 6 | 0,685 | a |
| BM172 | ctgtagctcaaacagggcact  gcaataccgccatgagagat | (GA)_23_ | 5 | 0,710 | a |
| BM197 | tggactggtcgatacgaagc  cccagaagattgagaacaccac | (GT)_8_ | 3 | 0,415 | a |
| BM210 | accactgcaatcctcatctttg  ccctcatcctccattcttatcg | (CT)_15_ | 7 | 0,684 | a |
| BMb174 | ttgaaacaaatcagaccctc  atacatagatgcaagagcga | (TTA)_10_ | 12 | 0,529 | b |
| BMb247 | atcctagggagtcatgaagg  agaattgtaaccacaccgac | (AT)_13_ | 5 | 0,686 | b |
| BMb267 | tgagcatcctctacttggtt  aatctcgcctctctctcttt | (GAA)_9_ | 3 | 0,403 | b |
| BMb469 | cattcatgtgaacctttcatt  attgtttggtttgtgcttct | (AGA)_10_ | 2 | 0,418 | b |
| BMb508 | ttgagacaaatgactcacca  cgtgttcctttaaacaatcc | (CTC)_7_ | 3 | 0,437 | b |
| BMb96 | cataaagcacgtcacttcaa  gccttggacactaccattt | (CA)_11_ | 4 | 0,645 | b |
| BMd12 | catcaacaaggacagcctca  gcagctggcgggtaaaacag | (AGC)_7_ | 2 | 0,389 | c |
| BMd20 | gttgccaccggtgataatct  gtgaggcaagaagccttcaa | (TA)_5_ | 4 | 0,499 | c |
| BMd22 | ggtcacttccggagcattc  cgggaaatggaagtcacagt | (TC)_6_ | 2 | 0,502 | c |
| BMd25 | gcagatcgcctactcacaaa  cgttgacgagaagcatcaag | (GAT)_6_ | 2 | 0,464 | c |
| BMd42 | tcatagaagatttgtggaagca  tgagacacgtacgaggctgtat | (AT)_5_ | 5 | 0,680 | c |
| BMd45 | ggttgggaagcctcatacag  atcttcgacccaccttgct | (AG)_5_ | 2 | 0,486 | c |
| BMd46 | ggctgacaacaactctgcac  ctggcataggttgctccttc | (TCT)_4_ | 2 | 0,409 | c |
| BMd47 | acctggtccctcaaaccaat  caatggagcaccaaagatca | (AT)_5_ | 2 | 0,414 | c |
| BMd53 | tgctgaccaaggaaattcag  ggaggaggcttaagcacaaa | (GTA)_5_ | 3 | 0,562 | c |
| GATS91 | gagtgcggaagcgagtagag  tccgtgttcctctgtctgtg | (GA)_17_ | 14 | 0,861 | a |
| PVag001 | caatcctctctctctcatttccaatc  gaccttgaagtcggtgtcgttt | (GA)_11_ | 5 | 0,661 | d |
| PVag003 | tcacgtacgagttgaatctcaggat  ggtgtcggagaggttaaggttg | (AG)_8_ | 4 | 0,454 | d |
| PVat007 | agttaaattatacgaggttagcctaaatc  cattcccttcacacattcaccg | (AT)_12_ | 19 | 0,885 | d |
| Pvctt001 | gagggtgtttcactattgtcactgc  ttcatggatggtggaggaacag | (CTT)_3_(T)_3_(CTT)_6_ | 4 | 0,509 | d |
| Average |  |  | 5.269 |  |  |
| Total |  |  | 137 |  |  |

N_a_ - No. of alleles in this study; H*_E_* - Gene Diversity

a - Gaitán-Solís et al 2002; b - Córdoba et al. 2010; c - Blair 2013; d - Yu et al. 2000.
