# Supplementary figures and images for: Genetic Diversity of Croatian Common Bean Landraces
Source: Front Plant Sci. 2017 Apr 20;8:604. doi: 10.3389/fpls.2017.00604 (PMC5397504; doi:10.3389/fpls.2017.00604)

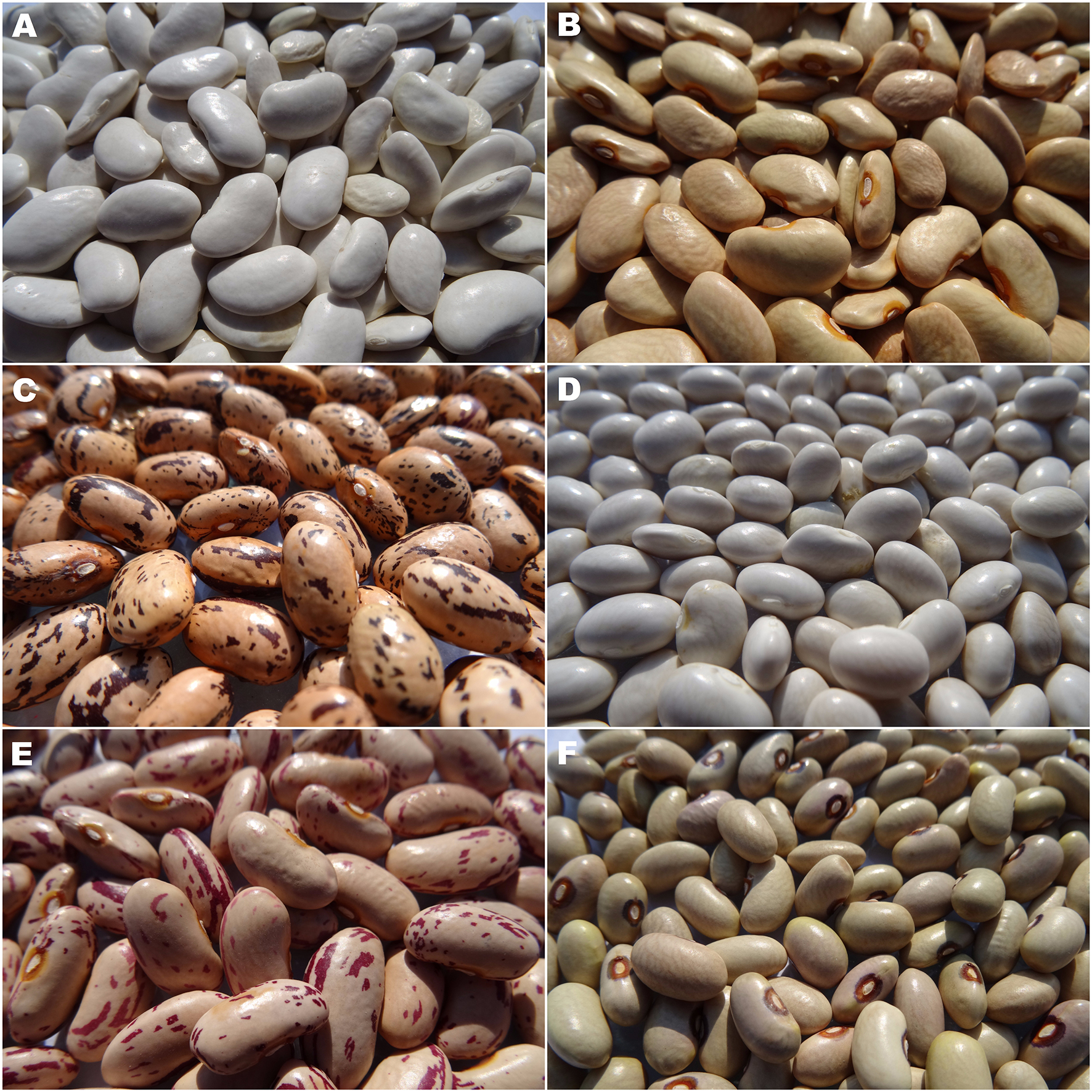

Supplement: Supplementary file 3 [file Image1.JPEG]
