# Supplementary material for: Genetic Diversity of Croatian Common Bean Landraces
Source: Front Plant Sci. 2017 Apr 20;8:604. doi: 10.3389/fpls.2017.00604 (PMC5397504; doi:10.3389/fpls.2017.00604)
Supplement: Supplementary file 4 [file DataSheet1.DOC]

Supp. Figure 1. Seeds of Croatian common bean accessions. The Mesoamerican group (cluster A / phaseolin type I): (**A**) Ph247, (**B**) Ph137; Andean group B (cluster B / phaseolin type II): (**C**) Ph400, (**D**) Ph184; Andean group C (cluster C / phaseolin type III): (**E**) Ph052, (**F**) Ph021
